# Malaria control and elimination in Kenya: economy-wide benefits and regional disparities

**DOI:** 10.1186/s12936-023-04505-6

**Published:** 2023-04-07

**Authors:** Zuhal Elnour, Harald Grethe, Khalid Siddig, Stephen Munga

**Affiliations:** 1 Humboldt-Universität zu Berlin, Berlin, Germany; 2 Agricultural Research Corporation, Wad Madani, Sudan; 3 International Food Policy Research Institute, Khartoum, Sudan; 4 University of Khartoum, Khartoum, Sudan; 5 Kenya Medical Research Institute, Kisumu, Kenya

**Keywords:** Malaria, Household welfare, Economic performance, CGE analysis, Human health, Public health policy, Kenya

## Abstract

**Background:**

Malaria remains a public health problem in Kenya despite several concerted control efforts. Empirical evidence regarding malaria effects in Kenya suggests that the disease imposes substantial economic costs, jeopardizing the achievement of sustainable development goals. The Kenya Malaria Strategy (2019–2023), which is currently being implemented, is one of several sequential malaria control and elimination strategies. The strategy targets reducing malaria incidences and deaths by 75% of the 2016 levels by 2023 through spending around Kenyan Shillings 61.9 billion over 5 years. This paper assesses the economy-wide implications of implementing this strategy.

**Methods:**

An economy-wide simulation model is calibrated to a comprehensive 2019 database for Kenya, considering different epidemiological zones. Two scenarios are simulated with the model. The first scenario (*GOVT*) simulates the annual costs of implementing the Kenya Malaria Strategy by increasing government expenditure on malaria control and elimination programmes. The second scenario (*LABOR*) reduces malaria incidences by 75% in all epidemiological malaria zones without accounting for the changes in government expenditure, which translates into rising the household labour endowment (benefits of the strategy).

**Results:**

Implementing the Kenya Malaria Strategy (2019–2023) enhances gross domestic product at the end of the strategy implementation period due to more available labour. In the short term, government health expenditure (direct malaria costs) increases significantly, which is critical in controlling and eliminating malaria. Expanding the health sector raises the demand for production factors, such as labour and capital. The prices for these factors rise, boosting producer and consumer prices of non-health-related products. Consequently, household welfare decreases during the strategy implementation period. In the long run, household labour endowment increases due to reduced malaria incidences and deaths (indirect malaria costs). However, the size of the effects varies across malaria epidemiological and agroecological zones depending on malaria prevalence and factor ownership.

**Conclusions:**

This paper provides policymakers with an ex-ante assessment of the implications of malaria control and elimination on household welfare across various malaria epidemiological zones. These insights assist in developing and implementing related policy measures that reduce the undesirable effects in the short run. Besides, the paper supports an economically beneficial long-term malaria control and elimination effect.

## Background

Malaria remains a public health problem in Kenya despite the scale-up of intervention tools [1]. Every year, nearly 6.7 million clinical cases of malaria are reported in Kenya, with 70% of the population being at risk of malaria [2]. It is estimated that approximately 4000 people die from malaria annually, most of them being children. Besides, malaria is responsible for 13–15% of outpatient consultations [3].

**Fig. 1:**
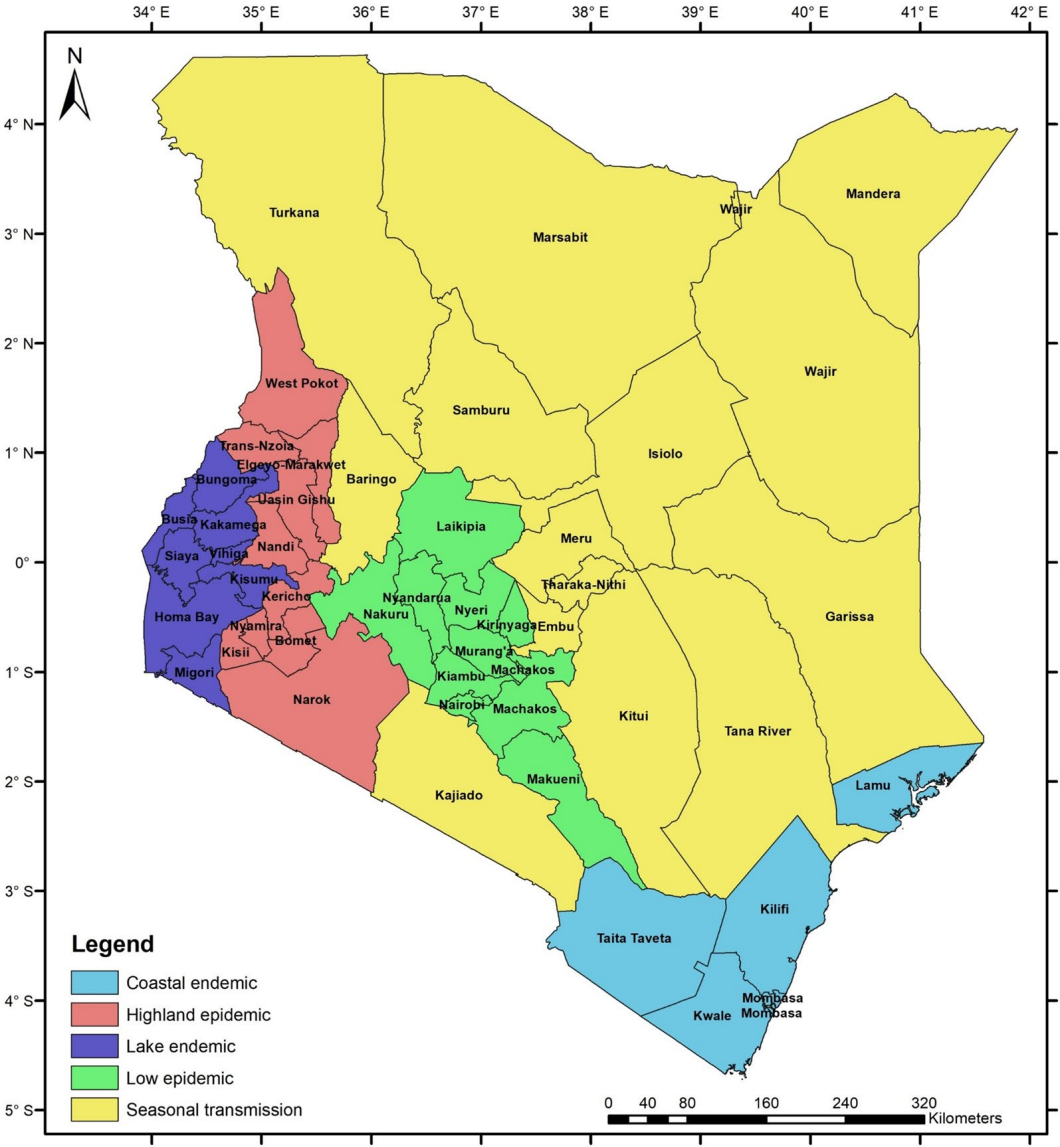
Malaria epidemiological map for Kenya. Source: Author's compilation based on [6]

Climate change and farming practices, such as deforestation, are expected to increase malaria incidence [4]. Malaria transmission and infection risk in Kenya are closely related to altitude, temperature, and rainfall patterns. Consequently, malaria prevalence varies considerably across seasons and regions [5, 6]. The country has been classified into five malaria epidemiological zones to address the varied risks, as shown in Fig. 1. These zones include: (a) coastal endemic, (b) lake endemic, (c) seasonal malaria transmission, (d) malaria epidemic-prone areas of western highlands and epidemic-prone areas, and (e) low-risk malaria areas.

Climatic conditions of the lake endemic and coastal endemic zones are suitable for high survival rates of the malaria vector. As a result, malaria transmission is intense throughout the year in these two regions, with high annual entomological inoculation rates. In 2015, malaria parasite prevalence was 27% and 8% in the lake endemic and coastal endemic zone, respectively [6].

Malaria transmission in the highland epidemic zone is seasonal, with a considerable annual variation. Transmission intensity increases under favourable climatic conditions for the malaria vector with sustained minimum temperatures around 18° C, which sustain vector breeding. In the regions where malaria occurs regularly, fatality rates during an epidemic can be up to 10 times greater than in the lake endemic and coastal endemic zones. In 2015, malaria parasite prevalence in this zone was 3% [6].

The seasonal malaria transmission zone includes the arid and semi-arid areas in Kenya's northern and south-eastern parts. It experiences short periods of intense malaria transmission during the rainy season. In 2015, malaria parasite prevalence was approximately 1% [6]. Under extreme climatic conditions like flooding, the zone is expected to experience malaria epidemics with high morbidity due to the low immunity of the population.

In the low-risk zone, malaria parasite prevalence in 2015 was less than 1% [6]. Low temperatures in this zone prevent the malaria parasite in the vector from completing the sporogonic cycle. Climate change, e.g., increasing temperatures and changes in the hydrological cycle, is likely to increase the areas suitable for malaria vector breeding, introducing malaria transmission in regions where it did not exist before [6].

Malaria morbidity and mortality comes along with costs of treatment, control and prevention, thus establishing a substantial economic burden. This results in reduced economic growth mainly by reducing the labour force. Moreover, malaria causes health care spending at private and public levels. Consequently, malaria restrains long-term economic growth and sustainable development.

The interest in economy-wide assessments of the economic costs of malaria and government health intervention policies has recently increased. Such assessments provide insight into the long-run economy-wide effects of malaria on economic growth and development and may support policymakers in adopting measures that would eliminate malaria. Such economy-wide assessment does not yet exist for Kenya.

This paper assesses the economy-wide impacts of the efforts toward malaria control and elimination in Kenya, referring to the objective of the Kenya Malaria Strategy (KMS) (2019–2023). Further, it measures the benefits of declining malaria incidences and deaths, while on the other hand, it captures the costs of increasing government expenditure on malaria treatment, control, prevention and eradication.

## Economic costs of malaria

**Fig. 2:**
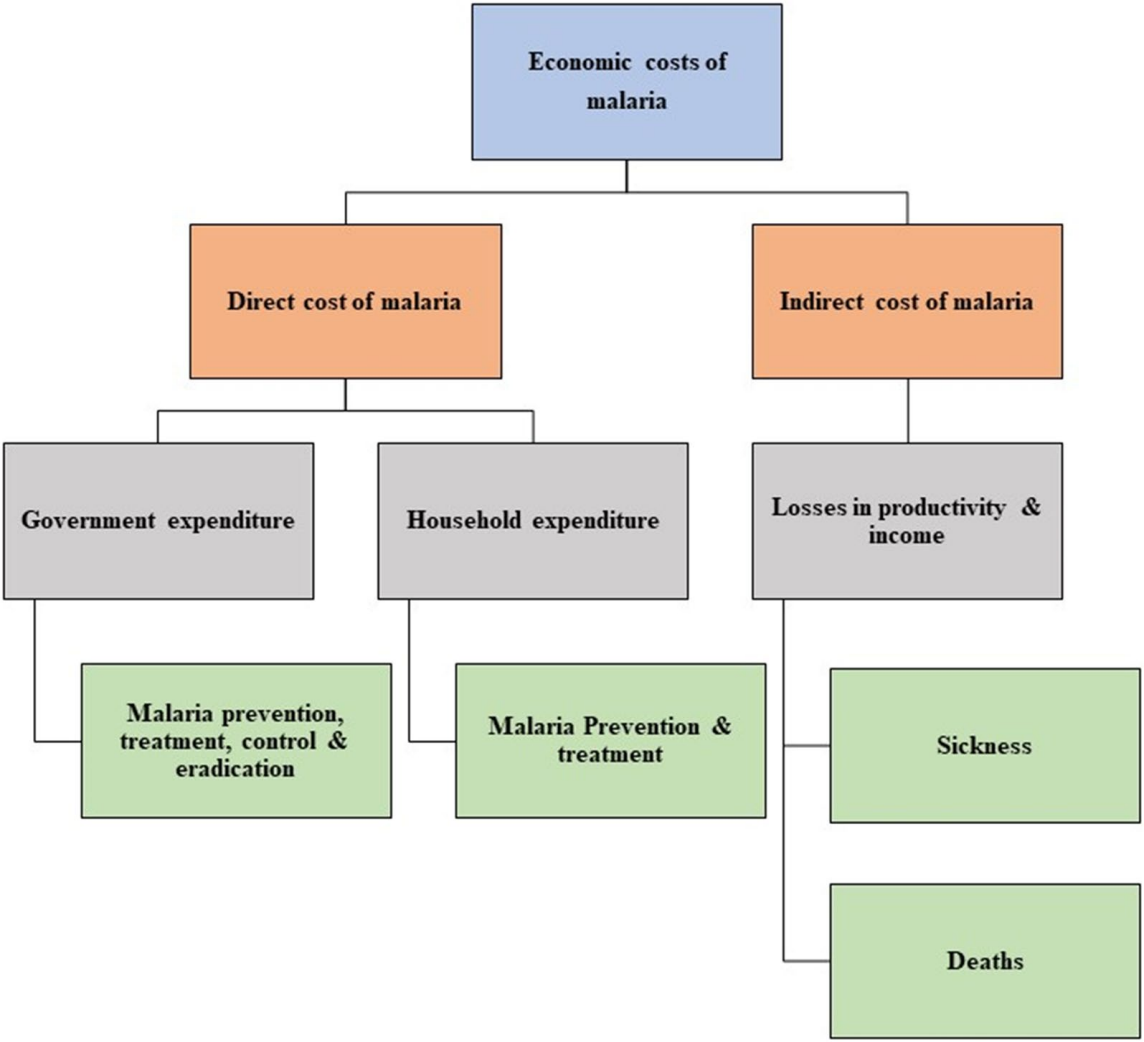
Types of economic costs of malaria. Source: Authors' compilation based on [4, 7, 9]

The economic costs of malaria can be classified into direct and indirect costs [4, 7, 8]. First, the direct malaria costs contain a combination of household and government expenditures on treating and preventing malaria, as illustrated in Fig. 2. Household expenditure on malaria treatment consists of individual or family spending on consultation fees, drugs, transport and the cost of subsistence at a distant health facility, and costs of accompanying family members during hospital stays [9]. Household expenditure on malaria-related prevention includes costs of buying preventive means, for instance, mosquito coils, aerosol sprays, bed nets and mosquito repellents [7]. According to the malaria-endemic degree, these means can be used differently across regions and counties.

Government expenditure on malaria-related treatment, control and prevention includes spending on maintaining health facilities and health care infrastructure, publicly managed vector control (e.g., insecticide-treated bed nets, indoor residual spraying, larviciding, community-wide campaigning), education and research. The indirect costs of malaria consist of losses in productivity or income due to illness or deaths [7, 9], as shown in Fig. 2. Losses due to sickness can be measured as the cost of lost workdays due to illness or caring for sick family members. In contrast, losses due to deaths can be calculated as discounted future lifetime earnings of those who die.

A study reviewing the empirical evidence on the indirect costs of malaria in Africa found considerable variation across the results of the reviewed studies, depending on the methods used for measuring and valuing time lost [7]. Indirect costs are often estimated using the wage method. This method defines the costs as the estimated time (workdays) lost multiplied by the working day wage (income). The time cost is determined as the sum of the opportunity cost of time foregone by the sick individual and the opportunity cost of healthy family member's time spent treating or caring for sick persons or accompanying them for treatment.

On average, time lost per episode for a sick adult ranges from 1 to 5 working days. The variation in time loss by episode depends on the prevalence of different malaria species, immunity levels in adults, accessibility to treatment services, type of economic activity, and mode of remuneration [7]. In Ethiopia, for example, the indirect costs of malaria account for approximately 78% of the total malaria costs incurred by private households [10].

Several empirical studies have assessed the direct economic costs of malaria in Kenya. One such study estimates that the total economic costs of malaria for children aged 5 years for the year 2009 is about US$ 251 million [8]. Total direct malaria costs represent 44% of total estimated costs. Malaria treatment expenditure accounts for 27%, of which private households cover about 68%. Indirect costs, including losses due to deaths, account for 57%.

The total cost of malaria hospitalization is approximately US$ 58 per person in Kenya, of which government costs represent 72% [4]. Total cost of malaria intermittent screening and treatment in school per child in 2010 is estimated at US$ 7 [11]. About 47% of these costs represent intervention costs, which comprises redeployment of existing resources, including health worker time and hospital vehicle use.

An empirical study that evaluated the effects of malaria on wage income in Kenya, concluded that a 10% increase in malaria prevalence reduced the monthly individual wage income by 3.3% to 3.8% [12]. Besides, total economic malaria costs represent (on average) 1% of total household income [13]. Moreover, nearly 170 million working days are lost annually due to malaria in Kenya [14].

Since 2004, The Kenyan government has implemented sequential Kenya malaria strategies to control and eliminate malaria [6]. Each strategy is developed based on the recommendations and evaluation of the malaria programme review of the previous strategy. The shared vision of these strategies is to free Kenya from malaria by directing and coordinating efforts through effective national and international partnerships. The current KMS (2019–2023) aims to reduce malaria incidence and deaths by at least 75% of the 2016 levels by 2023. To achieve this target, approximately Kenyan Shillings (Ksh) 61.9 billion will be spent in total over a period of 5 years on malaria control and elimination programmes.

## Depicting malaria effects in CGE models

Computable General Equilibrium (CGE) models depict the economy as a whole as a system of equations. These cover production (based on the standard assumption of profit maximization) and consumption (based on the standard assumption of utility maximization) of goods and mechanisms governing the economy as a whole such as the balance of government income and expenditure and a balanced exchange with the rest of the world. Such models allow for assessing the economic implications of complex and simultaneous changes in exogenous variables such as health expenditure and the labour force due to changes in human health. Especially, they can depict indirect effects on sectors and household groups. Such indirect effects, which are mediated through changes in product and factor prices, are often important in case of shocks affecting the economy as a whole. Several empirical studies attempt to estimate the economic costs of malaria, particularly in developing countries [7, 15–17]. Nevertheless, few CGE studies assess the economy-wide implications of malaria [18–21].

Climate change-induced changes in human health through malaria and other selected diseases are for example assessed using a global CGE model, namely the Global Trade Analysis Project (GTAP) model [21]. This study captures the effects of malaria on labour productivity and the demand for health care. Malaria affects labour productivity by changing mortality and morbidity. These effects are incorporated in the model as exogenous shocks. The changes in childhood mortality are determined by changes in the prevalence of vector-borne diseases resulting from a one-degree increase in global mean temperature. The relative annual loss of labour productivity equals the number of additional malaria deaths plus the additional years of working time lost, divided by the total population. In addition, malaria's effects on health care demand are captured in the model by changing the productivity of health services for private and public final demand.

Another CGE-based study evaluates the health and economy-wide impacts of malaria transmission in Ghana using an integrated epidemiological-demographic CGE model [20]. The epidemiological component depicts malaria infections and prevalence and calculates clinical outcomes for infected individuals. The clinical outcomes are used to estimate the effects of malaria on mortality and morbidity rates. These rates are applied in the demographic component for calculating changes in demographic structure due to malaria transmission. The demographic component classifies population by age group and gender type. It also includes international and interregional migration specifications. The CGE component is a recursive-dynamic model, which explicitly covers capital accumulation in different sectors over time. The key link between the three components is the determination of the labour force and ownership by population demographics based on two malaria-related morbidity rates. These are (a) the rate of female adults caring for sick children and (b) the rate of sick adults. Morbidity effects on the labour force are determined as the affected gender-specific working-age population group multiplied by gender-specific labour market participation rates, labour factor skill shares, rates of reduction in annual labour supply per malaria episode, and the average number of malaria episodes per person per year. The skill shares and participation rates are estimated using secondary data, i.e., labour force data from household surveys or the World Development Indicator database.

In contrast, the rate of reduction in annual labour supply per malaria episode is estimated endogenously in the CGE component. It is a function of intervention effective coverage rates and fixed morbidity rates associated with and without effective intervention treatment. Intervention effective coverage rates are defined by private and public malaria-related composite intervention commodities, underlying regional population levels in the case of prevention interventions and by number of uncomplicated episode cases.

This paper uses a static CGE model to assess the economy-wide impacts of the efforts to control and eliminate malaria in Kenya, following the recommendations from the Kenya Ministry of Health (KMoH) [6]. This policy benefits the Kenyan economy by decreasing the indirect costs of malaria, i.e., a reduction in malaria incidences and deaths. As a result, household labour endowment increases, which is incorporated in the model as an exogenous increase in household labour supply. In addition, implementing this policy increases the direct costs of malaria. Hence, the government expenditure on malaria control and elimination is expanded, which is depicted as an exogenous increase in government expenditure on health services.

## Methods

### Data

**Fig. 3:**
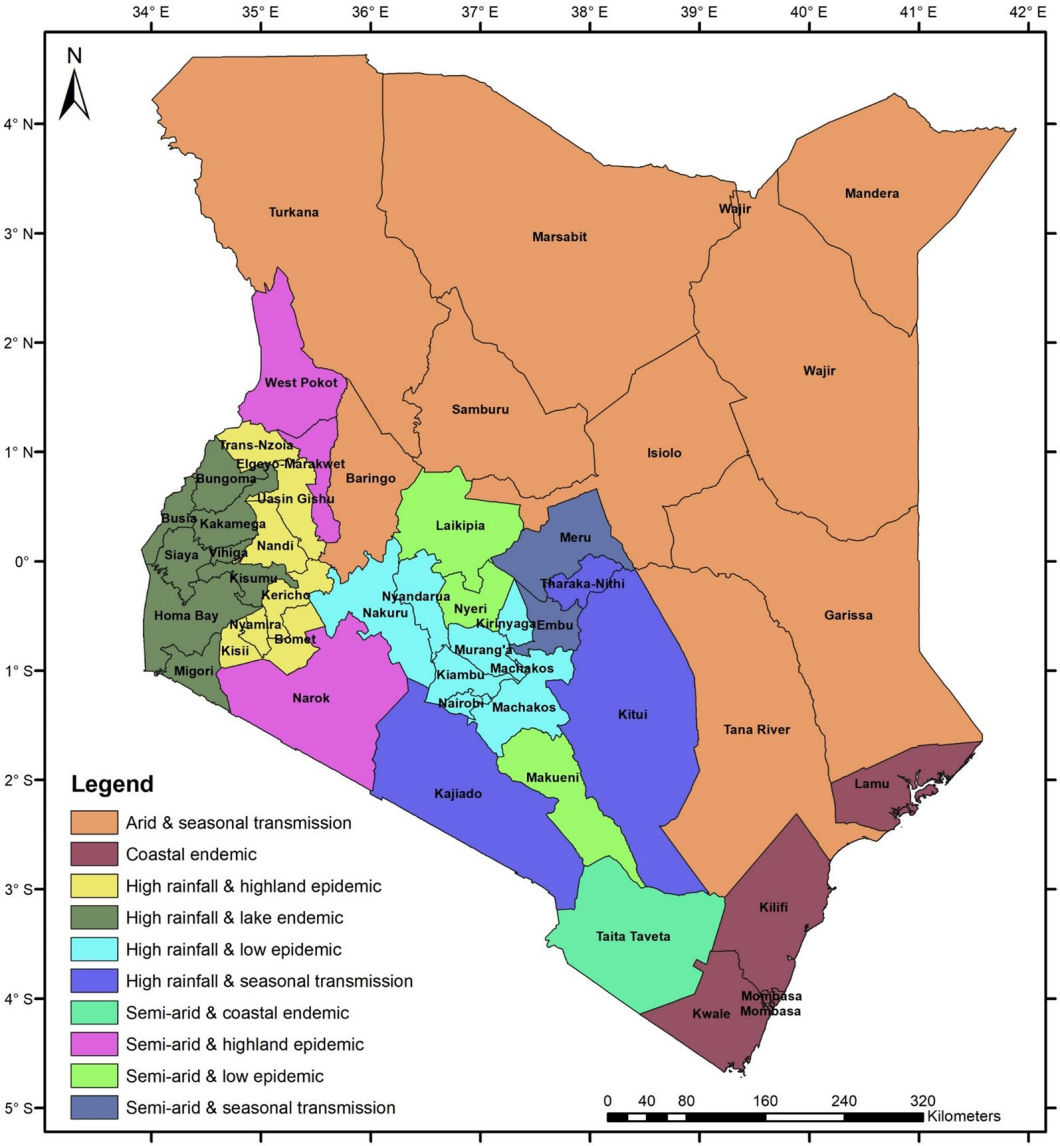
Kenyan malaria epidemiological and agroecological zones included in the 2019 SAM. Source: Author's compilation based on [6, 21]

A social accounting matrix (SAM), which is an economy-wide database, is developed for Kenya for the year 2019 [22]. The SAM contains data related to malaria epidemiological and agroecological zones, as shown in Fig. 3. These zones include: (a) arid and seasonal transmission, (b) coastal endemic, (c) high rainfall and highland epidemic, (d) high rainfall and lake endemic, (e) high rainfall and low epidemic, (f) high rainfall and seasonal transmission, (g) semi-arid and coastal zone, (h) semi-arid and seasonal transmission, (i) semi-arid and highland epidemic, and (j) semi-arid and low-risk epidemic.

The SAM is an update and extension of a 2017 SAM for Kenya [23] based on data from a 2019 SAM for Kenya [24] and domestic sources, including the Kenya National Bureau of Statistics (KNBS) [25], the KMOH [6], and the Central Bank of Kenya (CBoK) [26].

The 2019 micro-SAM for Kenya has 186 accounts representing the Kenyan economy. It identifies 51 production activities, of which 22 are agricultural, 19 are industrial, and the rest and the rest are services.

The SAM has 34 production factors: two capitals (agricultural and not), two lands (irrigated and not) and 30 labour categories. Labour is classified based on skill level into three categories: skilled, semi-skilled and unskilled labour. The three labour categories are regionalized based on the ten malaria epidemiological and agroecological zones (Fig. 3).

Households are disaggregated into 40 representative groups using three criteria: (a) ten malaria epidemiological and agroecological zones, (b) two residence places (rural and urban), and (c) two income levels (poor and non-poor). The remaining nine accounts consist of enterprises, trade and transport margins, government, four tax accounts (i.e., sales tax, import tax, production tax and income tax), one capital account (savings and investment), and the rest of the world account.

### Model

STAGE, a static CGE model, is used to assess the economy-wide impacts of malaria control and elimination in Kenya [27]. This type of model evaluates the effect of policy changes by comparing the equilibrium state of the economy before and after the reform. However, it does not show the process of the economy's transition from the initial equilibrium to the new equilibrium after a shock.

STAGE is based on standard microeconomic theory: productive activities maximize profits, and consumers maximize utility. Production is modelled as a three-level system of nested Constant Elasticity of Substitution (CES) and Leontief production functions. On the top level, activities combine aggregate primary production factors and aggregate intermediate inputs using a CES function. The different groups of production factors are aggregated using CES functions at different levels, while the intermediate input component is aggregated using a Leontief production function. Producers decide to sell their products either in the local market or the export market depending on relative prices according to a Constant Elasticity of Transformation (CET) function. Households supply their production factors to productive activities through factor markets (e.g., the labour market) against wages, which constitute a major source of their incomes. They spend their income on purchasing goods and services after paying taxes and making savings. The demand system is derived from a Stone-Geary utility function whereby households choose optimum mixes of commodities and services subject to their purchase prices and the constraints of preferences and income.

### Simulation design

Against the model base representing the Kenyan economy in 2019, two counterfactual scenarios (*GOVT* and *LABOR*) are developed to depict the effects of the KMS (2019–2023).

First, the *GOVT* scenario assesses the isolated effects of increasing the Kenya government expenditure on malaria control and elimination programs without considering the impacts on the labour force. The Kenyan government plans to spend around Ksh 12.4 billion annually over 5 years. This policy increases the total government expenditure on health services by 7% annually. This additional expenditure represents 0.9% of total government expenditure and 0.1% of current Gross Domestic Product (GDP). This scenario depicts a typical year during the "investment phase" of the strategy.

Second, the *LABOR* scenario simulates the effects of reducing malaria incidences by 75% in all epidemiological malaria zones without accounting for the changes in government expenditure. This reduction is expected to happen by the end of the policy implementation period. This scenario thus depicts a typical year during the "pay-off phase" of the strategy, which comprises the benefits of reduced malaria after the increased government expenditure in *GOVT*. The *LABOR* scenario translates the reduction in malaria into increasing working days by boosting household labour endowment, as explained in Table 1, based on a set of assumptions. These assumptions are developed using a database and reports for Kenya from Demographic and Health Surveys (DHS) produced by the United States Agency for International Development (USAID).

**Table 1:** Effects of malaria control and elimination strategy on labour services supplied by households per region. Source: Author's calculation based on [28, 31]

		1	2	3	4	5	6	7	8	9
Zone	Household group	Malaria prevalence across different household groups (%)			Malaria prevalence across zones (%)	Average national Malaria prevalence (%)	Reduction in malaria Incidences due to Implementing malaria strategy (2019–2023) (%)	Changes in household labour endowment due to implementing malaria strategy (2019–2023) (%)		
		Skilled	Semi-skilled	Unskilled				Skilled	Semi-skilled	Unskilled
Lake endemic	Rural poor	2.98	5.76	6.48	27.00	8.00	75.00	7.54	14.59	16.39
	Rural non-poor	1.40	1.61	2.60	27.00	8.00	75.00	3.55	4.07	6.58
	Urban poor	0.08	0.40	1.07	27.00	8.00	75.00	0.21	1.02	2.70
	Urban non-poor	0.03	0.03	0.19	27.00	8.00	75.00	0.07	0.08	0.48
Coastal endemic	Rural poor	2.98	5.76	6.48	8.00	8.00	75.00	2.24	4.32	4.86
	Rural non-poor	1.40	1.61	2.60	8.00	8.00	75.00	1.05	1.21	1.95
	Urban poor	0.08	0.40	1.07	8.00	8.00	75.00	0.06	0.30	0.80
	Urban non-poor	0.03	0.03	0.19	8.00	8.00	75.00	0.02	0.03	0.14
Highland epidemic	Rural poor	2.98	5.76	6.48	3.00	8.00	75.00	0.84	1.62	1.82
	Rural non-poor	1.40	1.61	2.60	3.00	8.00	75.00	0.39	0.45	0.73
	Urban poor	0.08	0.40	1.07	3.00	8.00	75.00	0.02	0.11	0.30
	Urban non-poor	0.03	0.03	0.19	3.00	8.00	75.00	0.01	0.01	0.05
Seasonal transmission	Rural poor	2.98	5.76	6.48	1.00	8.00	75.00	0.28	0.54	0.61
	Rural non-poor	1.40	1.61	2.60	1.00	8.00	75.00	0.13	0.15	0.24
	Urban poor	0.08	0.40	1.07	1.00	8.00	75.00	0.01	0.04	0.10
	Urban non-poor	0.03	0.03	0.19	1.00	8.00	75.00	0.00	0.00	0.02
Low risk area	Rural poor	2.98	5.76	6.48	0.50	8.00	75.00	0.14	0.27	0.30
	Rural non-poor	1.40	1.61	2.60	0.50	8.00	75.00	0.07	0.08	0.12
	Urban poor	0.08	0.40	1.07	0.50	8.00	75.00	0.00	0.02	0.05
	Urban non-poor	0.03	0.03	0.19	0.50	8.00	75.00	0.00	0.00	0.01

Malaria prevalence across different household groups in Kenya is presented in Table 2. Numbers in this table are calculated using the malaria indicator survey database for the year 2020 [28]. The table shows that rural (poor and non-poor) households lose more due to malaria than urban (poor and non-poor) households. This can be attributed to the availability and easy access to health services in urban areas [29]. Skilled households are less affected by malaria compared to semi-skilled and unskilled household groups (Table 2). This can be attributed to the access of skilled households to knowledge on prevention methods and incomes able to afford the costs of malaria treatment and prevention means [29].

**Table 2:** Malaria prevalence across different household groups for the year 2020 in Kenya. Source: Author's calculation based on [27]

	Skilled labour	Semi-skilled labour	Unskilled labour	Total
Rural poor households	2.98	5.76	6.48	5.42
Rural non-poor households	1.40	1.61	2.60	1.36
Urban poor households	0.08	0.40	1.07	0.47
Urban non-poor households	0.03	0.03	0.19	0.08

The changes in household labour endowment due to implementing the KMS (2019–2023) are calculated in Table 1. The relative malaria prevalence across household groups is assumed to be similar across different malaria epidemiological zones (columns 1–3). Malaria prevalence across different malaria epidemiological zones (column 4) and malaria prevalence at the national level (column 5) is obtained from the Kenya malaria indicator survey report for the year 2015 [30]. Reduction in malaria prevalence is obtained from the current implemented strategy documentation (column 6) [6].

Based on the above information, changes in household labour endowment are estimated (columns 7–9), as shown in Table 1. For instance, rural poor skilled households in the lake endemic zone lose 7.54% of total working days due to sickness or taking care of a sick family member. This figure is calculated by first multiplying malaria prevalence in the corresponding household group (2.98%) by the ratio of malaria prevalence in the lake endemic zone (27%) over the national malaria prevalence average of 8%. The resulting figure is multiplied by 75%, generating the reduction in lost working days due to malaria at the end of the KMS implementation period.

The differences across labour categories in the increase in labour service availability in response to malaria control and elimination (Table 3) are associated with regional and household differentiation. This can be explained by regional variation in skill composition of the labour force. Besides, the scenario considers differences in effects on skill level categories (see Table 2), which result from household groups being affected differently due to socioeconomic characteristics (e.g., poor/non-poor). As a result, the provision of labour services increases most for unskilled labour, the most affected labour category by malaria in Kenya.

**Table 3:** Total labour supply in the base and *LABOR* scenario (million persons and % change). Source: Author's calculation

Labour category	Initial year (million persons)	*LABOR* scenario (million persons)	Increase from base (%)
Skilled	6958.09	6979.27	0.30
Semi-skilled	9186.31	9328.45	1.55
Unskilled	1958.63	2004.35	2.33
Total	18,103.02	18,312.06	1.15

### Closure rules

The scenarios are implemented under the following closure rules. These closure rules reflect the main characteristics of the Kenyan economy. First, the macro closure includes a savings-driven neoclassical approach. This closure describes the relationship between total saving and total investment in the economy. Savings rates of households and enterprises are assumed to be constant, allowing total savings to change according to income changes. Consequently, total investment spending changes to accommodate changes in total savings. Second, the government closure holds the value of government consumption expenditure constant at its initial level. Besides, government savings are fixed in absolute terms and household income taxes vary to clear the government account. Third, the factor market closure assumes full employment of factors in all markets. In addition, all factors are assumed to be mobile across sectors, with fixed overall factor supply and wage rates clearing the market. Fourth, the small country assumption is used to fix world market prices. Besides, the external balance (foreign savings) is kept constant by a flexible exchange rate. Last, the CPI is the model numéraire.

## Results

This section reports simulation results as changes in values of model variables relative to their values in the reference scenario. After presenting the effects on factor prices and quantities of domestic production, it discusses the effects on household welfare. The section ends with a sensitivity analysis showing how different choices of financing instruments affect the simulation results.

### Factor prices

**Fig. 4:**
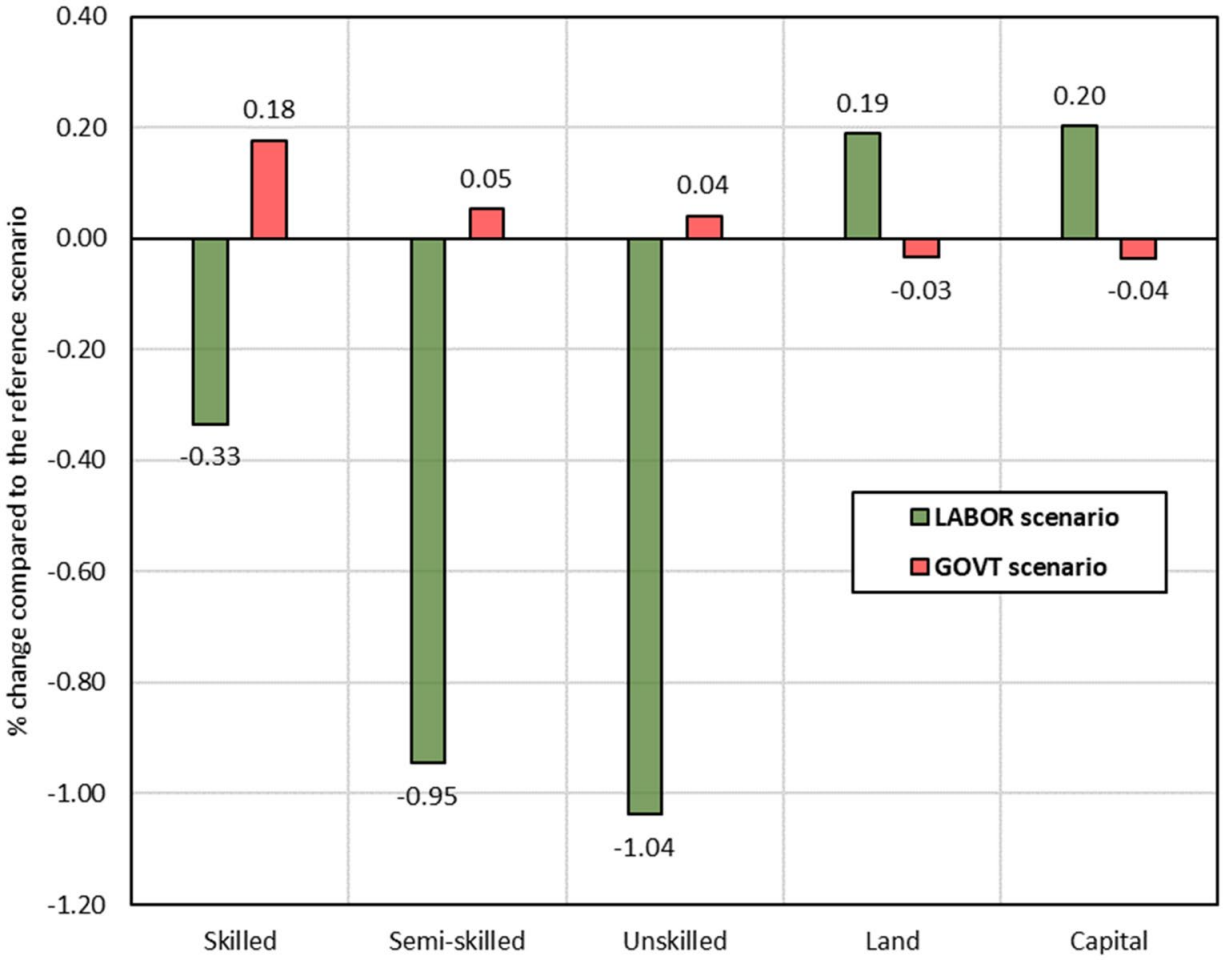
Effects on factor prices (% change compared to the reference scenario). Source: Author's calculations based on simulation results

The effects of counterfactual scenarios on factor prices are illustrated in Fig. 4. The *GOVT* scenario captures the annual cost of the KMS (2019–2023), expanding government expenditure on malaria control and elimination programs. This scenario increases labour wages, which is strongest for skilled labour, and is caused by the expansion of the health sector being labour-intensive and especially skilled labour-intensive sector. The expansion of the health sector is funded by increasing taxes and results in overall lower domestic consumption and production. As a result, total demand for capital and land decreases, driving down their prices.

The *LABOR* scenario depicts the benefit of the KMS (2019–2023) in terms of increased labour supply. As a result, wages decline relative to the reference scenario. The wage for unskilled labour drops more than for other labour categories because of its higher increase in supply (shock structure). Simultaneously, prices for complementary factors (i.e., capital and land) increase, driven by a relative supply shortage and increase in demand.

### Domestic production

**Fig. 5:**
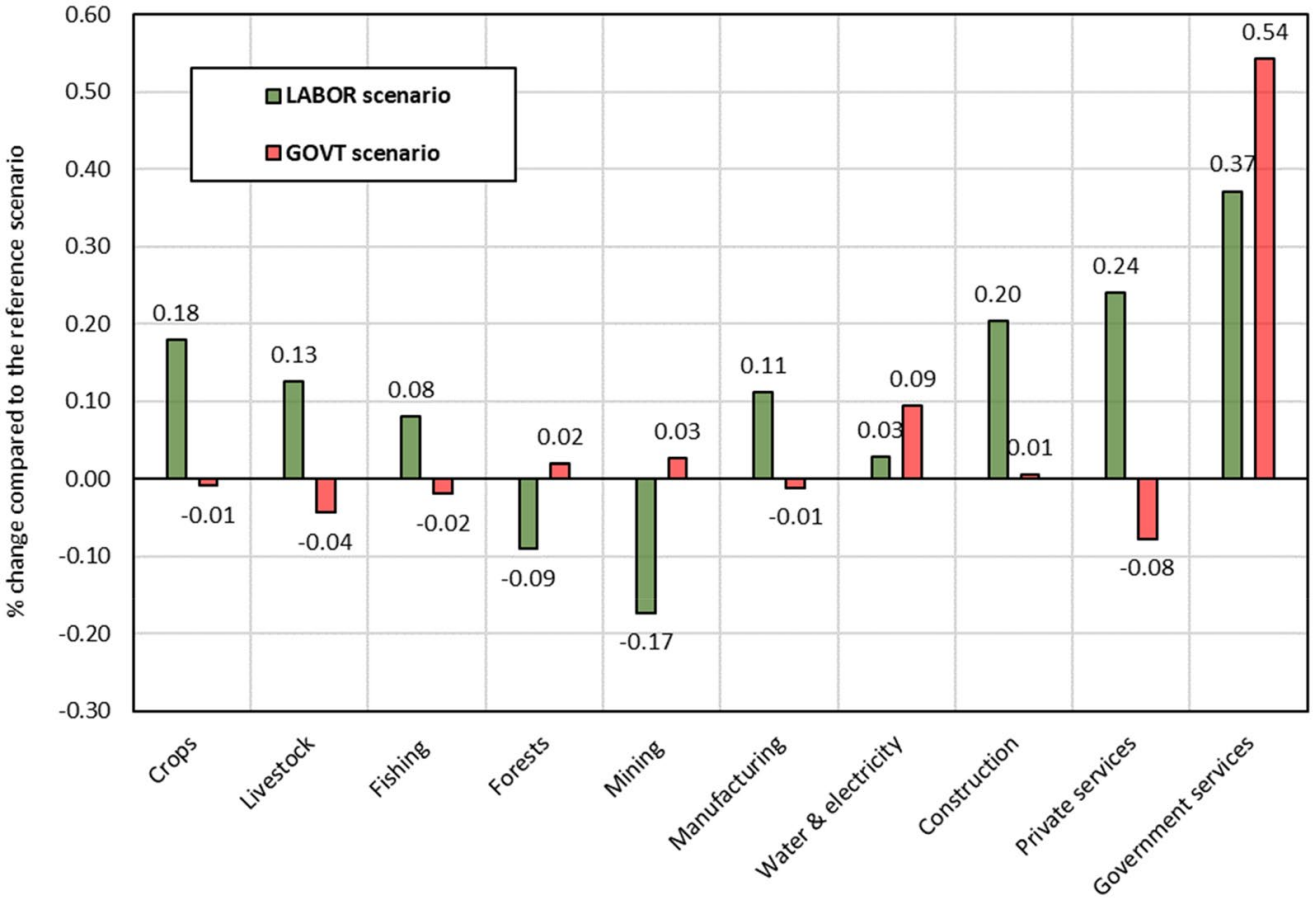
Effects on quantities of sectoral domestic production (% change compared to the reference scenario). Source: Author's calculations based on simulation results

Effects of the two scenarios on quantities of domestic production vary across sectors, as shown in Fig. 5. The variation across sectors is explained by differences in their cost structure. The *GOVT* scenario boosts government services production by about 0.5%, driven by expanding expenditures on malaria control and elimination program. However, the expansion of health services is financed by increasing household tax payments. This increase reduces the domestic demand and production of crops, livestock, fishing, and private services. Production of manufacturing, water, electricity, and construction increases because they are important intermediate inputs in the health sector. In contrast, the reduction in capital price and the growth of construction increases slightly production of forests and mining, which are capital intensive sectors and significant intermediate inputs in construction sector.

Under the *LABOR* scenario, reducing malaria prevalence and incidence drives up sectoral domestic production in Kenya driven by increased total labour supply and lower production costs. Production of crops, construction, and services (i.e., trade and administration) increase more than in other sectors, as shown in Fig. 5, because these sectors have the most prominent labour share from the lake and coastal endemic zones, the two regions that benefit the most from malaria elimination. Production of forests and mining decreases under this scenario due to increased prices of non-labour factors, which are used intensively by these two sectors.

### Household welfare

**Fig. 6:**
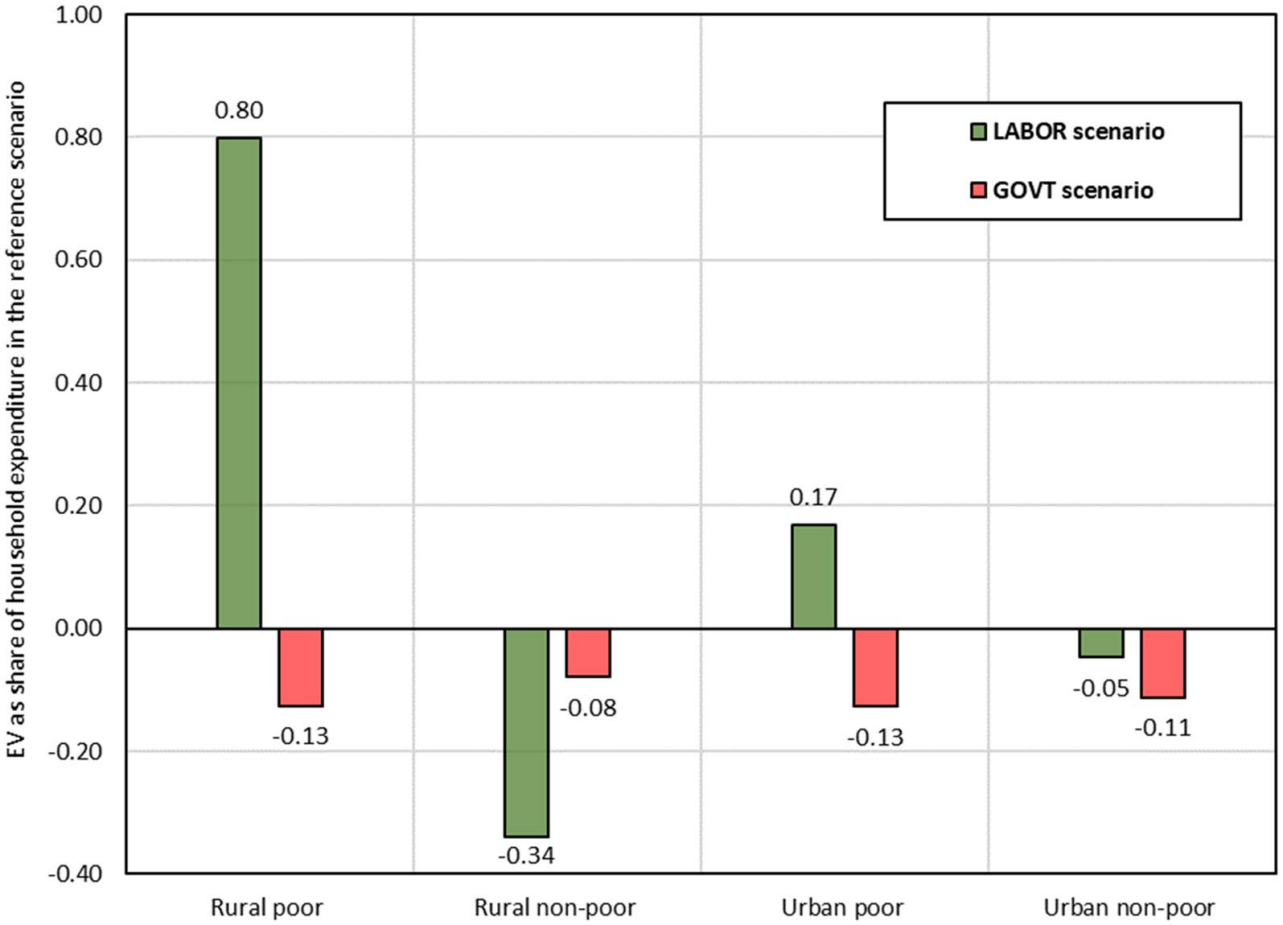
Effects on household welfare (EV as a share of household expenditure in the base situation). Source: Author's calculations based on simulation results

Figure 6 presents the effects of the two scenarios on household welfare, which are measured using the Equivalent Variation (EV). The welfare effects vary across scenarios and representative household groups. This variation can be explained by differences in income sources, consumption patterns, and tax payments.

The *GOVT* scenario expands health services, which increases its demand for production factors, as illustrated earlier in Fig. 4. In contrast, production costs of non-health sectors increase, driving up their producer and consumer prices. Moreover, because the implemented policy is financed by increasing household income tax payments, disposable household income decreases. Consequently, the welfare of all households drops. Additionally, poor households in rural and urban areas lose more than non-poor households in relative terms (Fig. 6), which can be attributed to their lower income shares from skilled labour, for which wages increase most (Fig. 4).

In the *LABOR* scenario, controlling and eliminating malaria increases labour supply and lowers wages. This boosts household income because the increase in labour supply is more significant than the reduction in wage rates. It also boosts domestic production because it reduces the cost of production for most sectors. Subsequently, total household welfare increases (Fig. 6).

Figure 6 illustrates that rural household welfare increases more than urban household welfare in the *LABOR* scenario. This difference is driven by the growth in labour supply, which has a higher share in rural income than in urban income. Furthermore, this scenario boosts poor household income more than non-poor household income, due to higher malaria incidences amongst poor households (shock structure). Rural non-poor households lose because of high-income tax payments and a low increase in their factor incomes, particularly labour incomes.

**Fig. 7:**
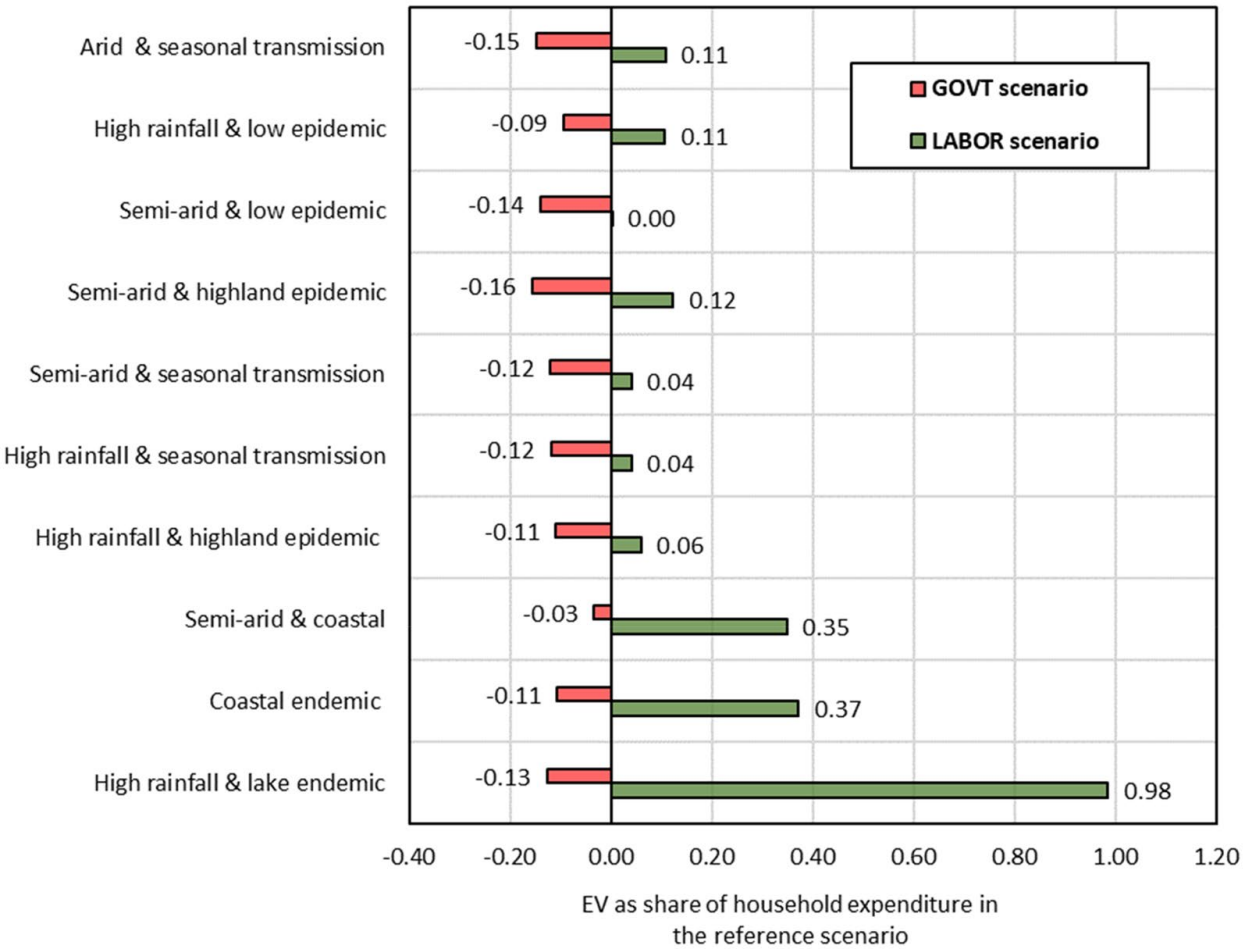
Effects on household welfare across malaria epidemiological and agroecological zones. Source: Author's calculations based on simulation results

The effects on household welfare across malaria epidemiological and agroecological zones are presented in Fig. 7. It shows that the *GOVT* scenario decreases household welfare in all malaria epidemiological and agroecological zones due to higher income tax payments and product prices compared to the base situation. In the *LABOR* scenario, lake and coastal endemic households benefit more than others due to the increase in their factor income driven by high labor supply growth. In contrast, the welfare of households in the highland epidemic, low epidemic, arid and seasonal transmission zones improve mainly due to the increased income from the complementary factors of land and capital.

### Sensitivity analysis

A sensitivity analysis is conducted to assess the choice of instrument to finance the malaria control and elimination interventions under the *GOVT* scenario. It examines the model's robustness and the sensitivity of results to variations in the closure rules and assumptions. In addition to the household income tax, two alternative instruments are chosen to finance government expenditure on health services (*GOVT* scenario): a sales tax and foreign transfers to the government.

**Fig. 8:**
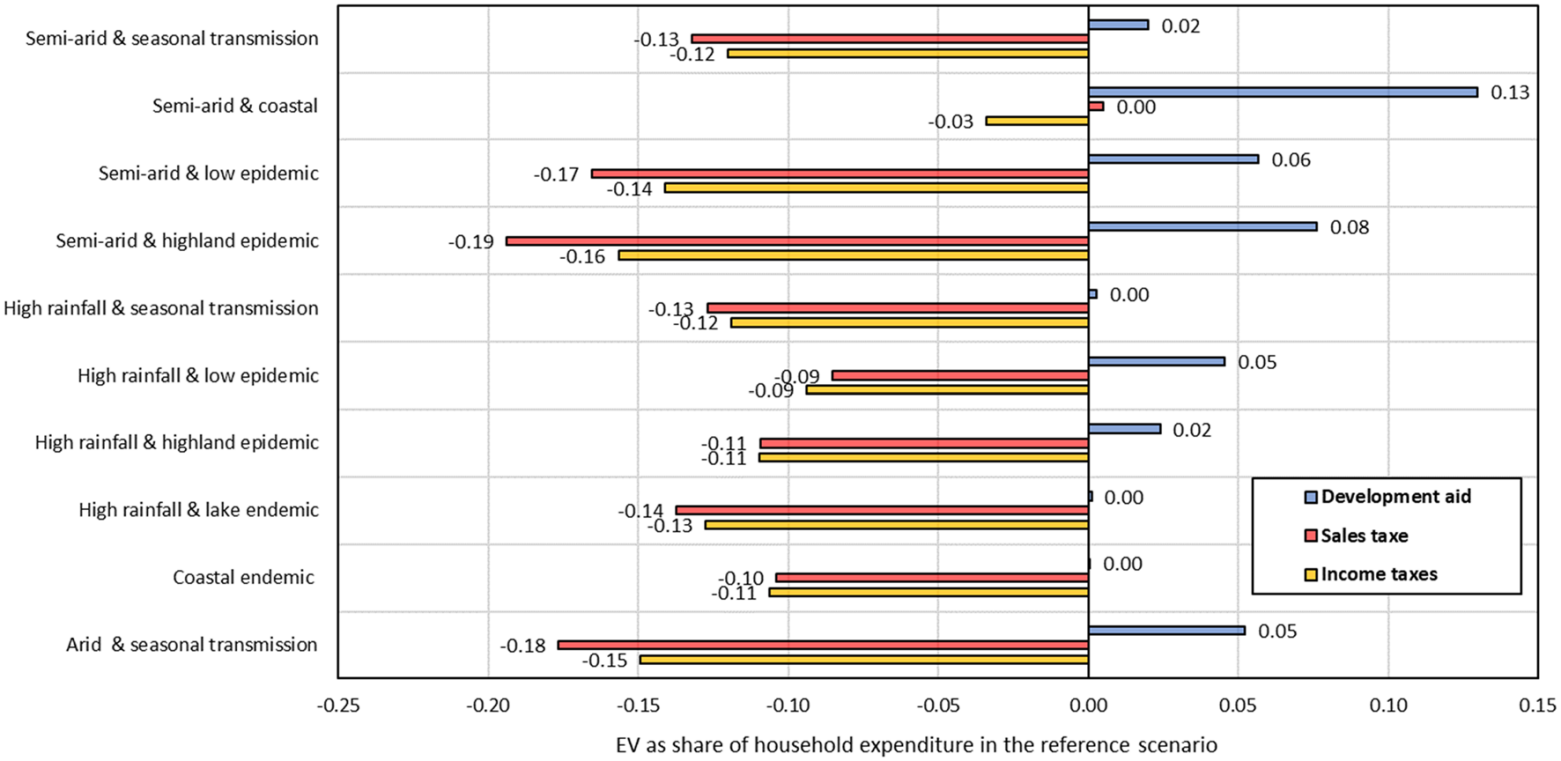
Sensitivity analysis of different instruments for financing the implemented policy on household welfare. Source: Author's calculations based on simulation results

Figure 8 illustrates the sensitivity analysis results, taking household welfare as an example. It shows that the magnitude of the results varies only slightly among different tax instruments. Under the foreign transfer to government instrument, all households benefit slightly. This is plausible as domestic taxpayers do not need to fund government interventions.

## Discussion

This study found that implementing the Kenya Malaria Strategy 2019–2023 influences the Kenyan economy in two different and often opposite ways. On the one hand, it increases household labour endowment due to reduced malaria prevalence, which positively influences the economy by increasing domestic production. At the aggregate level, agriculture and services, both labour-intensive sectors, would benefit more than industry (non-labour-intensive). At the sectoral level, sectors that use labour intensively in high endemic zones, such as the crop sector, would benefit more than other sectors. The effects on household welfare would be positive, though they vary across malaria epidemiological and agroecological zones due to the differences in malaria relevance and labour ownership. Consequently, the real GDP increase is driven by the growth in the total labour force.

Implementing this strategy requires expanding government expenditure on health services. Expanding health services increases the demand for production factors and thus factor prices. Producer and consumer prices for products from non-health sectors increase. As a result, the consumption patterns of domestic consumers change. However, the increase in consumer prices exceeds the increase in household income. Hence, household welfare declines. In contrast, the real GDP increases slightly, driven by the significant growth in government consumption.

The sensitivity analysis regarding the financing instruments for expanding health services shows that tax-based financing instruments reduce the welfare of all household categories. In contrast, financing the strategy through increasing foreign transfers to government benefits all households slightly.

Finally, some suggestions for future research in the field are highlighted, which are related to five shortcomings of this study. First, the study focuses on incorporating the benefits and costs of the malaria control and elimination strategy in a comparative static model setup, not considering the time path throughout the implementation process. Such an analysis does not adequately depict the time path from short- to long-run effects. For instance, the adverse effects of expanding government consumption at the expense of private consumption and investment demand are expected to fade out after the end of the spending period. The growth of household labour endowment due to reduced malaria prevalence phases in stepwise and is a permanent effect.

Second, the first objective of the KMS (2019–2023) is to protect 100% of people living in endemic malaria zones through access to appropriate preventive interventions by 2023 [6]. Consequently, the Kenyan government plans to spend 67% of additional malaria control and elimination expenditure on scaling up malaria prevention and control interventions, e.g., distribution of long-lasting insecticidal nets, indoor residual spraying, larval source management, and establishment of documents for malaria vector control. The rest of the additional elimination expenditure (33%) is planned to be spent on activities related to malaria treatment, elimination, and management. Nevertheless, expenditure on malaria control and prevention programs is aggregated with other health services in our model database. This implies that additional malaria control and elimination expenditures cannot be depicted with adequate detail. A detailed database covering the individual health-related sectors would enhance the analysis.

Third, the study does not incorporate the effects on mortality rates, which prevents capturing changes in population demographics. This would require linking the CGE model to a demographic model to analyse the impacts of demographic and health condition changes on the labour force, as suggested by Jensen et al. [18]. Future research could benefit from such a model combination.

Fourth, the study does not capture the negative effects of malaria on children's education and school outcomes. This kind of assessment could require including different types of educational cycles in the model and its database and linking labour force via educational outcomes to trace the changes in the educational outcomes. It also would require model specifications such as dynamic CGE model, which considers time dimension and assumes that behaviours of firms and households are derived from intra- and intertemporal optimization. Last, neither variations in labour income due to malaria control and elimination according to the type of employment nor gender were integrated. For instance, people employed in non-permanent jobs, e.g., self-employed in agricultural sectors, may lose more income in high malaria endemic zones than those with permanent jobs because of losses in payment for absent working days due to malaria. To depict such effects would require disaggregating labour according to employment type in each zone.

## Conclusions

This paper applied an economy-wide (CGE) model for assessing the economy-wide implications of malaria control and elimination in Kenya, considering regional malaria disparities. Two scenarios are developed based on the costs and benefits of implementing the KMS (2019–2023). The first scenario captures the annual costs of decreasing malaria prevalence by increasing government expenditure on malaria control, treatment and prevention programs (the direct malaria costs), which would prevail for 5 years. The second scenario depicts the benefits of implementing the KMS by increasing household labour endowment (reducing the indirect malaria costs). The results show that applying the KMS (2019–2023) enhances overall economic performance as measured by growth in GDP at the end of the strategy implementation compared to the reference scenario without the implantation of the strategy. In terms of private household welfare, more than 10 years would be needed to compensate for the investment period through a higher labour endowment.

Although the paper does not capture the specific time path of costs and benefits, it provides policymakers with an ex-ante assessment of the implications of malaria control and elimination on household welfare across various epidemiological malaria zones. These insights could assist in developing and implementing related policy measures that reduce the negative effects in the short term, e.g., increasing subsidies or social transfers for those who are more negatively affected by the strategy in the short run.

## Figures and Tables

**Fig. 9:**
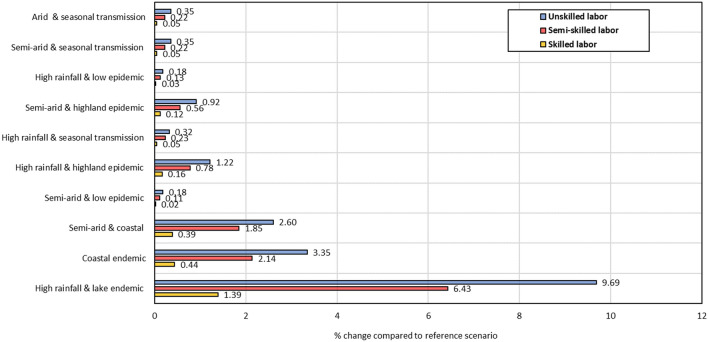
Changes in labour supplies in the *LABOR* scenario (% change compared to the reference scenario). Source: Author's calculations based on simulation results

**Fig. 10:**
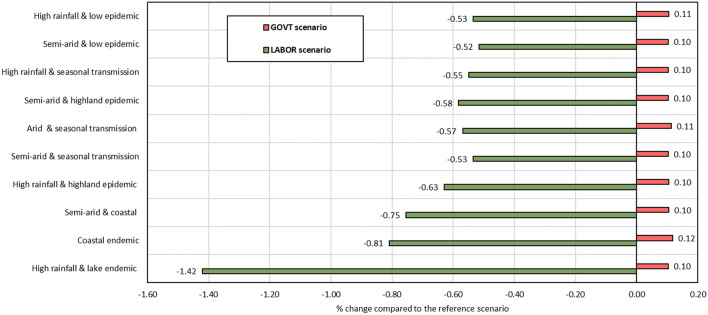
Effects on labour price across malaria epidemiological and agroecological zones, Source: Author's calculations based on simulation results

**Fig. 11:**
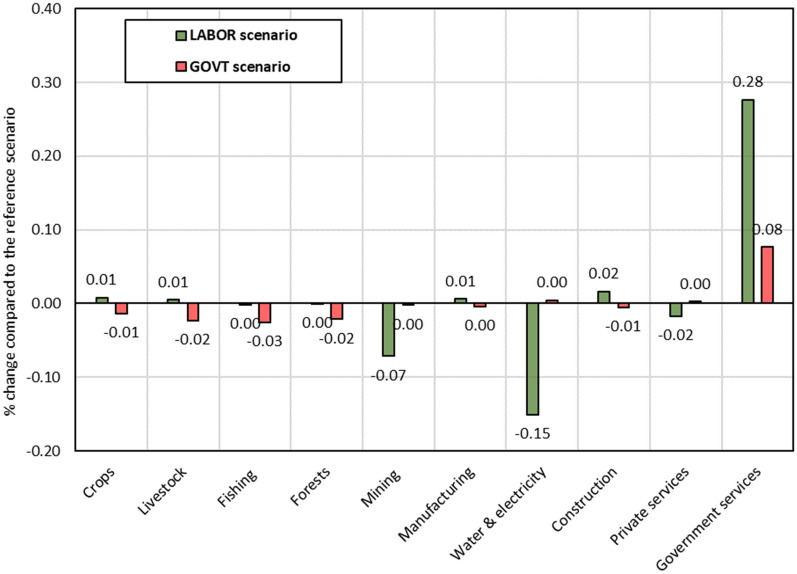
Effects on domestic producer prices (% change compared to the reference scenario). Source: Author's calculations based on simulation results

**Fig. 12:**
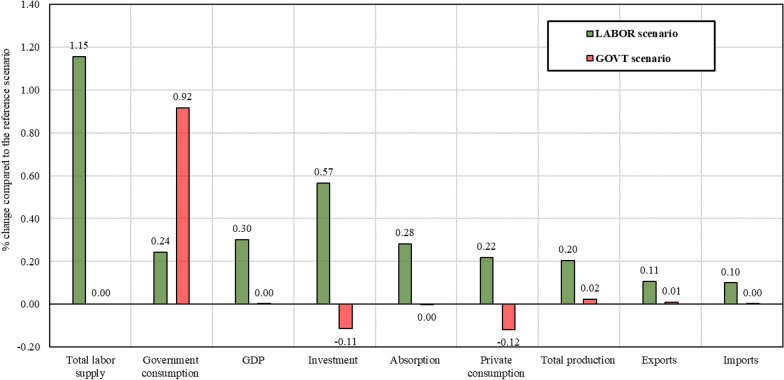
Effects on aggregate economy-wide indicators (% change compared to the reference scenario). Source: Author's calculations based on simulation results

## Data Availability

The datasets generated and analysed are publicly available.

## Appendices

### Appendix A

See Fig. 9.

### Appendix B

See Fig. 10.

### Appendix C

See Fig. 11.

### Appendix D

See Fig. 12.

### Appendix E

See Table 4.

**Table 4:** Effects on quantities of agricultural production by sector (% change compared to the reference scenario). Source: Author's calculations based on simulation results

	Labor	Government	% Total production	% Sectoral production
Cereals	0.17	− 0.06	1.73	10.44
Roots & tubers	0.16	− 0.04	0.03	0.19
Pulses & oil seeds	0.21	− 0.02	0.09	0.53
Fruits & vegetables	0.12	− 0.04	1.95	11.73
Sugarcane	0.22	− 0.07	0.31	1.84
Coffee	0.15	− 0.03	0.51	3.06
Tea	0.41	− 0.06	3.14	18.92
Cotton	0.09	− 0.02	0.77	4.63
Tobacco	− 0.79	0.16	0.47	2.86
Others crops	− 0.76	0.12	2.08	12.50
Beef	0.11	− 0.05	1.11	6.71
Dairy	0.17	− 0.05	0.54	3.24
Poultry & eggs	0.08	− 0.03	0.24	1.45
Other livestock	0.13	− 0.04	1.85	11.10
Fishing	0.18	− 0.02	0.59	3.52
Forestry	− 0.09	0.02	1.21	7.28
Total	**0.08**	− **0.01**	**16.62**	**100.00**

### Appendix F

See Table 5.

**Table 5:** Effects on macroeconomic indicators across different scenarios of government expenditure (% change compared to base). Source: Author's calculations based on simulation results

Macroeconomic indicators	Increase in expenditure on malaria control and elimination programs by 5% (alternative scenario)	Increase in expenditure on malaria control and elimination programs by 10% (planned annual investment by NMCP)	Increase in expenditure on malaria control and elimination programs by 20% (alternative scenario)
Total labor supply	0.000	0.000	0.000
Government consumption	0.458	0.915	1.830
GDP	0.000	0.000	0.001
Investment	− 0.057	− 0.114	− 0.228
Absorption	0.000	0.000	− 0.001
Private consumption	− 0.059	− 0.118	− 0.235

### Appendix G

See Table 6.

**Table 6:** Effects on macroeconomic indicators across different scenarios that simulate the effects of malaria reduction (% change compared to base). Source: Author's calculations based on simulation results

Macroeconomic indicators	Increase in household labour endowment due to reduction in malaria effects by 1% (alternative scenario)	Increase in household labour endowment due to reduction in malaria effects by 75% (desired level by NMCP)	Increase in household labour endowment due to reduction in malaria effects by 50% (alternative scenario)
Total labour supply	0.015	1.155	0.770
Government consumption	0.003	0.243	0.162
GDP	0.004	0.301	0.201
Investment	0.008	0.566	0.378
Absorption	0.004	0.282	0.188
Private consumption	0.003	0.218	0.145

### Appendix H

See Table 7.

**Table 7:** Distribution of Kenyan counties across malaria epidemiological and agroecological zones. **Source****:** Author's calculations based on [6, 32]

No	Zone andf county	No	Zone and county
1.0	Arid and seasonal transmission	6.0	High rainfall and low epidemic
1.1	Baringo	6.1	Kiambu
1.2	Garissa	6.2	Kirinyaga
1.3	Isiolo	6.3	Nairobi
1.4	Mandera	6.4	Machakos
1.5	Marsabit	6.5	Murang'a
1.6	Samburu	6.6	Nakuru
1.7	Tana River	6.7	Nyandarua
1.8	Turkana		
1.9	Wajir		
2.0	Coastal endemic	7.0	High rainfall & seasonal transmission
2.1	Kwale	7.1	Kajiado
2.2	Kilifi	7.2	Kitui
2.3	Lamu	7.3	Tharaka-Nithi
2.4	Mombasa		
3.0	High rainfall & highland epidemic	8.0	Semi-arid & coastal
3.1	Bomet	8.1	Taita–Taveta
3.2	Kericho		
3.3	Kisii		
3.4	Nandi		
3.5	Nyamira		
3.6	Trans Nzoia		
3.7	Uasin Gishu		
4.0	High rainfall & lake endemic	9.0	Semi-arid & seasonal transmission
4.1	Bungoma	9.1	Embu
4.2	Busia	9.2	Meru
4.3	Homa Bay		
4.4	Kakamega		
4.5	Kisumu		
4.6	Migori		
4.7	Siaya		
4.8	Vihiga		
5.0	Semi-arid & low epidemic	10.0	Semi-arid & highland epidemic
5.1	Laikipia	10.1	Narok
5.2	Makueni	10.2	West Pokot
5.3	Nyeri	10.3	Elgeyo-Marakwet

## Abbreviations

CBoK: Central Bank of Kenya
CES: Constant Elasticity of Substitution
CET: Constant Elasticity of Transformation
CGE: Computable General Equilibrium
DHS: Demographic and Health Surveys
EV: Equivalent Variation
GDP: Gross domestic product
GTAP: Global Trade Analysis Project
KMS: Kenya Malaria Strategy
KMoH: Kenya Ministry of Health
KNBS: Kenya National Bureau of Statistics
Ksh: Kenyan Shillings
SAM: Social accounting matrix
USAID: United States Agency for International Development
WHO: World Health Organization
